# Symbiotic microbial population composition of *Apolygus lucorum* under temperature and pesticide pressures

**DOI:** 10.3389/fmicb.2024.1485708

**Published:** 2024-12-04

**Authors:** Mengxin Ma, Hui Xue, Xiangzhen Zhu, Li Wang, Lin Niu, Junyu Luo, Jinjie Cui, Xueke Gao

**Affiliations:** ^1^Research Base of Zhengzhou University, State Key Laboratory of Cotton Bio-Breeding and Integrated Utilization, Institute of Cotton Research, Chinese Academy of Agricultural Sciences, Anyang, China; ^2^State Key Laboratory of Cotton Bio-Breeding and Integrated Utilization, School of Agricultural Sciences, Zhengzhou University, Zhengzhou, China; ^3^College of Plant Science and Technology, Huazhong Agricultural University, Wuhan, China

**Keywords:** *Apolygus lucorum*, imidacloprid, temperature effect, symbiotic bacteria, pest control

## Abstract

Insect population control using pesticides faces new challenges as global temperatures change. Symbiotic bacteria of insects play a key role in insect resistance to pesticides, and these symbiotic bacteria themselves are sensitive to the effects of temperature changes. *Apolygus lucorum*, a sucking pest, survives in a wide range of temperatures (15°C–35°C), and is presently controlled predominantly using the pesticide imidacloprid. Here, we investigated the effects of temperature and imidacloprid on *A. lucorum* microbial population composition using 16S rRNA sequencing. We found that the application of imidacloprid in high-temperature environments led to an increase in the species diversity of bacteria in the body of *A. lucorum.* High temperatures may disrupt the symbiotic relationship between certain bacteria and *A. lucorum*, such as *Cedecea neteri*. High temperatures led to a decrease in the abundance of *Cedecea neteri*. *Agathobaculum butyriciproducens*, *Advenella migardefenensis*, and *Akkermansia muciniphila* were very sensitive to temperature and were strongly affected by temperature changes. Microorganisms that were greatly affected by the concentration of imidacloprid in the community include *Aeromonas caviae* and *Akkermansia muciniphila*. The aim of this study is to reveal the dynamics and diversity of symbiotic bacteria of *A. lucorum* treated with imidacloprid at a range of temperatures. These results provide insight into new strategies for pest control in a changing climate.

## 1 Introduction

The mirid bug *Apolygus lucorum* belongs to Miridae (Hemiptera) and is an important worldwide agricultural pest that can survive in a wide temperature range (15°C–35°C) (Lu et al., 2012; Tan et al., 2018). In China, the population of *A. lucorum* significantly increased after the adoption of genetically modified cotton (Lu et al., 2008). Research has shown that if remedial measures are not taken in a timely manner, mirid and mirid related damage to cotton production in China will continue to increase with the expansion of *Bacillus thuringiensis* (Bt) cotton planting area (Wu et al., 2002). The insecticide imidacloprid (neonicotinoid) is widely used against sucking pest insects, including *A. lucorum* (Tan et al., 2012). Insect resistance to imidacloprid is usually affected by temperature, where in most cases, it exhibits a positive temperature effect meaning that with increased temperature, insects exhibit increased sensitivity. Research shows that the toxicity of imidacloprid to non target insects *Eisenia Andrei* and *Folsomia Candidai* increases with increasing temperature (Bandeira et al., 2020). It has been reported that the toxicity of imidacloprid to *A. lucorum* in Cangzhou, Hebei Province, also increased with increasing temperature (Ma et al., 2012).

The world is experiencing unprecedented challenges due to global climate changes, with rising temperatures driving biodiversity loss and habitat deterioration (Bálint et al., 2011). In the face of global climate change, an important research priority is to better understand how insect pests respond and adapt to rising temperatures in conjunction with changing pest management practices (Potts et al., 2010; Moran and Alexander, 2014). Findings suggest possible links between warmer temperatures, more abundant pests, and frequent insecticide applications which disrupt habitats (Crossley et al., 2024).

Previous research has shown that changes in temperature affect known pest mitigation strategies. For example, five populations of *A. lucorum* collected from cotton crops at different locations in China were evaluated for resistance to common insecticide lambda-cyhalothrin. The results showed that the population collected from Shandong Province exhibited 30-fold greater resistance to lambda-cyhalothrin (Zhen and Gao, 2016). Additionally, a moderate increase of temperature to 31°C enhanced the tolerance of pest *Bemisia tabaci* (Gennadius) to insecticide thiamethoxam (Guo et al., 2018). There are several reasons why pest control may be difficult in higher temperature conditions. Besides the direct impacts of global warming on the biology of insect, temperature increases may also alter the environmental fate of pesticides in the terrestrial compartments (Noyes et al., 2009). For instance, pesticide solubility, degradation, and volitization may increase under high temperatures (Römbke et al., 2007; Broznić and Milin, 2012). Also, temperature can influence the toxicity (or efficacy) of an insecticide (DeLorenzo et al., 2006). Furthermore, temperature can have a profound impact on the mutualistic interactions between insects and their symbionts (Kikuchi et al., 2016; Corbin et al., 2017).

There are a large number of microbial symbionts in insects, including those obtained from the surrounding environment and food (Luo et al., 2021; Coolen et al., 2022). *Apolygus lucorum* has a large number of symbiotic bacteria at different developmental stages that are essential for its resistance and growth and development (Xue et al., 2023). Previous research has noted that imidacloprid resistance and symbiotic bacteria have a relationship. For example, aphids infected with *Hamiltolella* exhibit lower sensitivity to insecticides including imidacloprid. The bacterial density in the aphids infected with *Hamiltolella* slightly decreased, but then increased sharply after treatment with different insecticides (Li et al., 2021). In *Bemisia tabaci*, imidacloprid had a significant effect on strains with dual infections of *Rickettsia arsenopsis* and *Wolbachia arsenopsis*, which also carried a high number of symbiotic bacteria (Ghanim and Kontsedalov, 2009). Temperature can affect the host, symbiotes, and their relationships as observed by their behavior and physiological activities. The thermal environment experienced by microorganisms interacting with the host is influenced by the host’s behavior and physiological activities, such as temperature regulation behavior and microbial environment selection (Arnold et al., 2019; Truitt et al., 2018; Hague et al., 2020). Symbiotic bacteria are also sensitive to environmental factors, thereby changing the host’s resistance to imidacloprid. Studies have shown that increasing temperature can have a negative impact on the symbiosis between *Nilaparavata lugens* and bacteria, leading to increased adverse reactions of the host to imidacloprid (Zhang et al., 2021).Therefore, a complex bidirectional relationship exists between insects and symbiotes that can tolerate and adapt to different thermal environments (Iltis et al., 2022). The response of pest symbiotic microorganisms to temperature is also an important cause of altered pesticide efficacy.

In this study, we use the important agricultural pest *A. lucorum* to examine the effect of temperature and imidacloprid on symbiotic microbial population dynamics and diversity and *A. lucorum* insecticide susceptibility. We used16S rRNA sequencing to explore the relationship of microorganisms to temperature and imidacloprid on *A. lucorum*. Our results reveal that temperature and imidacloprid concentration affect the microbial community composition and abundance on *A. lucorum*. *Aeromonas caviae* and *Akkermansia muciniphila* have a high correlation with imidacloprid concentration, and the overall community is greatly affected by imidacloprid concentration. *A. caviae* and *A. muciniphila* may be key microorganisms that determine the resistance of *A. lucorum* to imidacloprid in high temperature conditions. Our findings support that host-bacterial symbiosis interactions affect insect sensitivity to pesticides. The results presented here regarding the *A. lucorum* microbiome support the development of microbially based strategies for the management of insect pests (Crotti et al., 2012).

## 2 Materials and methods

### 2.1 Insect rearing and maintenance

*Apolygus lucorum* adults were originally collected from the cotton field in Zhengzhou, Henan Province, China. *Apolygus lucorum* were reared in an Artificial Climate Incubator and maintained at 25 ± 1°C and 55 ± 5% relative humidity, with a 16 h: 8 h (light: dark) photoperiod. Nymphs of *A. lucorum* were fed with green pods and corn, and adults were provided with 10% sucrose solution as a supplement.

### 2.2 Sample collection, DNA extraction, and 16S rRNA amplification sequencing

We treat the *A. lucorum* by feeding them green pods soaked in different concentrations of imidacloprid at different temperatures. Firstly, we selected the 3rd instar nymphs of *A. lucorum* and pre-treated them in an artificial climate incubator at 15°C (denoted as T15), 25°C (denoted as T25), and 35°C (denoted as T35) for 24 h. During this incubation they were fed normally. Next, we thoroughly cleaned the green pods, select undamaged and moderately sized green pods, soak them in a 0.1% Tween-80 aqueous solution, denoted as T, 18.24 mg/L (LC15 at 25°C) imidacloprid, denoted as L, 50.71 mg/L (LC30 at 25°C) imidacloprid, denoted as H, for 30 s, and fed it to the pre-treated *A. lucorum* nymphs. Then we sampled live insects after 48 h. In total, green pods were sampled from three temperatures (T15, T25, and T35), and treatments with tween, low imidacloprid concentration, or high imidacloprid concentration (T, L, and H). This is a total of 9 treatments, with three replicates for each treatment. The labeling method is temperature treatment + imidacloprid concentration treatment + replicate number. For example, T15H1 denotes a green pod sample that was incubated at 15°C in 50.71 mg/L imidacloprid from the first group of replicates. Surface-sterilized insects were used to analyze the changes in the composition of symbiotic bacteria. To remove microbial contaminants on the surface of insects prior to PCR amplification and sequencing, each sample was soaked in 70% ethanol for 5 min, followed by 10% bleach for 30 s, and then rinsed with sterile ultrapure water. The mirids were frozen by liquid nitrogen and stored at −80°C for further experiment.

DNA was extracted from whole insects using TIANamp Genomic DNA Kit (Tiangen Biotech (Beijing) Co., Ltd.) according to the manufacturer’s instructions. DNA was quantified using a Qubit Fluorometer with a Qubit dsDNA BR Assay Kit (Thermofisher, Massachusetts, United States), and the quality was assessed by running an aliquot on 1% (mass fraction) agarose gel. The full-length 16S rRNA gene were amplified with primer pairs 27F: AGRGTTTGATYNTGGCTCAG and 1492R: TASGGHTACCTTGTTASGACTT. Both the forward and reverse 16S primers were tailed with sample-specific PacBio barcode sequences to allow for multiplexed sequencing. We chose to use barcoded primers because this reduces chimera formation as compared to the alternative protocol in which primers are added in a second PCR reaction. The KOD One PCR Master Mix (TOYOBOLife Science) was used to perform 25 cycles of PCR amplification, with initial denaturation at 95°C for 2 min, followed by 25 cycles of denaturation at 98°C for 10s, annealing at 55°C for 30 s, and extension at 72°C for 1 min 30 s, and a final step at 72°C for 2 min. The final PCR amplicons were purified with VAHTSTM DNA Clean Beads (Vazyme, Nanjing, China) and quantified using the Qubit dsDNA HS Assay Kit and Qubit 3.0 Fluorometer (Invitrogen, Thermo Fisher Scientific, Oregon, United States). After individual quantification of each PCR, amplicons were pooled in equal amounts. SMRTbell libraries were prepared from the amplified DNA by SMRTbell Express Template Prep Kit 2.0 according to the manufacturer’s instructions (Pacific Biosciences). Purified SMRTbell libraries from the pooled and barcoded samples were sequenced on a PacBio Sequel II platform (Beijing Biomarker Technologies Co., Ltd., Beijing, China) using Sequel II binding kit 2.0.

### 2.3 Bioinformatic analysis

High quality sequencing reads with more than 97% similarity thresholds were allocated to one operational taxonomic unit (OTU) using USEARCH (version 10.0). Taxonomy annotation of the OTUs/ASVs was performed based on the Naive Bayes classifier in QIIME2 using the SILVA database (release 138.1) and with a minimum confidence threshold of 70%.

Alpha diversity refers to the species diversity, or richness, within a specific region or ecosystem. Alpha index analysis software: QIIME2.^1^ Commonly used indicators to measure microbial abundance include Chao1 index and ACE index. Complimentary measurements for measuring microbial diversity include Shannon wiener diversity index and Simpson diversity index. The dilution curve is calculated using the relative ratio of known OTUs in the measured rRNA sequence to calculate the expected number of OTUs when extracting n (where n is less than the total number of measured reads) reads. Then, a curve is drawn based on the expected number of OTUs corresponding to a set of n values (usually a set of arithmetic sequences less than the total number of sequences).

In order to select whether to use an RDA or CCA model, we first, used specific sample abundance data for DCA analysis, and checked the size of the first axis of length of gradient in the analysis results. If it was greater than 4.0, CCA was selected. If it was between 3.0 and 4.0, both RDA and CCA could be selected. If it was less than 3.0, RDA results were better than CCA. The R language vegan (v2.3) package was used to analyze and plot RDA or CCA while the online platform BMKCloud^2^ was used to analyze the sequencing data.

PICRUSt2 is a software that predicts sample functional abundance based on the abundance of marker gene sequences in the sample. Using PICRUSt2, we aligned the feature sequence (16S rRNA) with the reference sequence of the Integrated Microbial Genomes database to construct an evolutionary tree, identify the “nearest species” of the feature sequence, and predict the genetic information of unknown species based on the known species’ gene types and abundance information. PICRUSt2 can integrate the 16S rRNA gene sequence into COG^3^ and use the COG database to make predictions.

### 2.4 Statistical analysis

Differences were compared using the Tukey’s HSD following ANOVA. Different letters indicate significant differences.

## 3 Results

### 3.1 Sequencing quality analysis

A total of 169,395 sequences were obtained through high-throughput sequencing of 16S rRNA from *A. lucorum*. The results of sample sequencing data processing are shown in Supplementary Table S1. Based on a similarity level of 97%, a total of 180 OTUs were obtained, belonging to 8 phyla, 11 classes, 33 orders, 60 families, 117 genera, and 168 species (Supplementary Table S2). In order to determine microbial community richness, we randomly selected a certain number of sequences from the sample, counted the number of species represented by these sequences, and constructed a dilution curve based on the number of sequences and species (Supplementary Figure S1). As the number of sampling sequences increases the sample dilution curve of OTU plateaus, thus we observe that the diversity of *A. lucorum* microbial community has effectively reached saturation. This comprehensive evaluation of the richness of the microbial community indicates that the 16S rRNA samples reflects the types and numbers of *A. lucorum* microorganisms under different treatments.

### 3.2 Comparative analysis of microbiota diversity indices among different temperature in *A. lucorum* with imidacloprid

**Figure 1:**
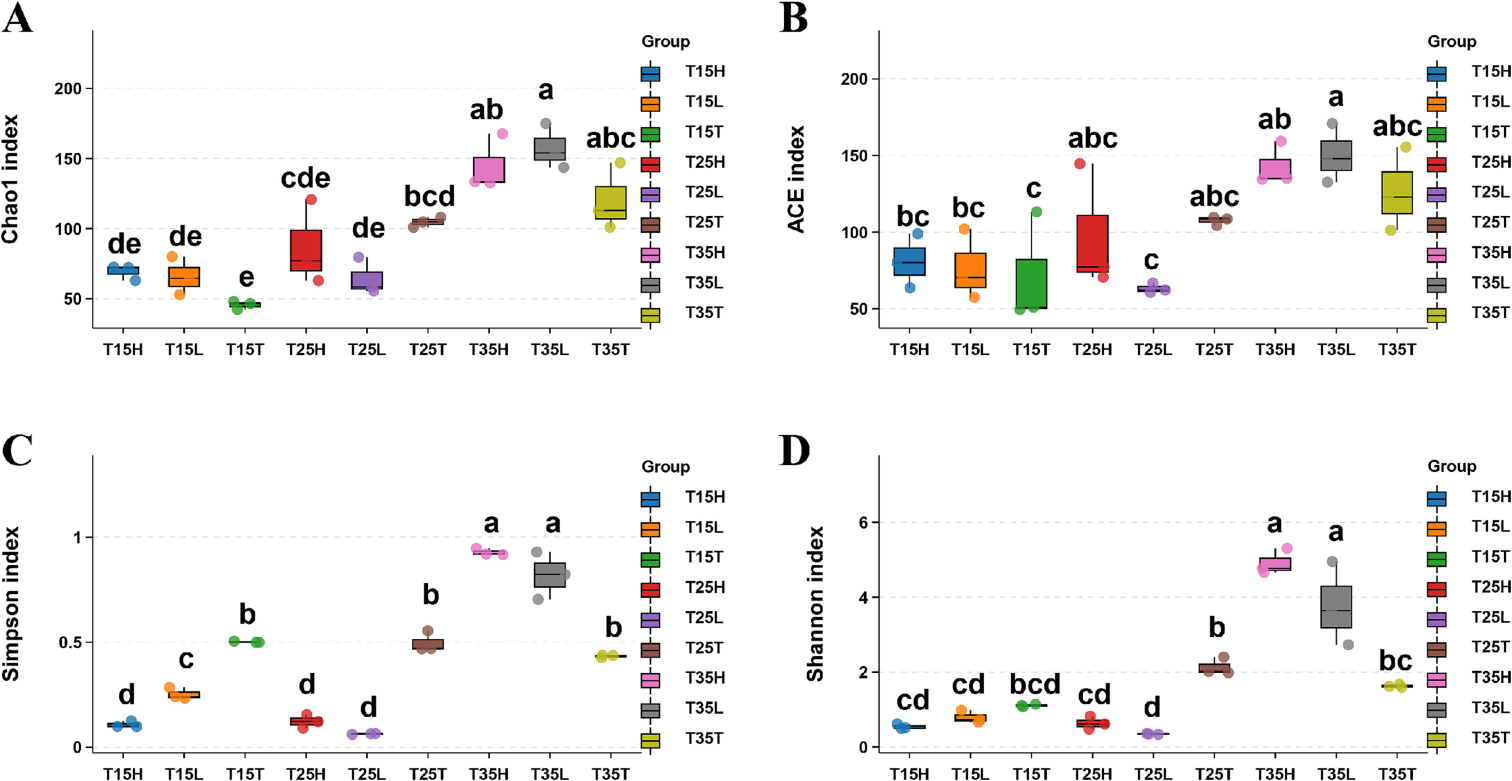
Sequencing analysis of 16S rRNA gene amplicons of *Apolygus lucorum* in different samples. **(A)** Chao1 index, **(B)** ACE index, **(C)** Simpson’ diversity, **(D)** Shannon’s diversity. Letters indicate differences based on Tukey’s HSD following ANOVA.

We used four metrics to explore alpha diversity: Chao1, Simpson, ACE, and Shannon indices (Figures 1A–D and Supplementary Table S3). We surveyed three temperature treatment conditions (T15, T25, and T35) and three concentrations of imidacloprid treatments (T, L, and H).

When the concentration of imidacloprid is the same, high temperature treatment (T35) produced higher indices of Chao1 and ACE than those treated with medium and low temperature (T15, T25). That is, the species richness of microorganisms in *A. lucorum* treated with high temperature is higher than the species richness of microorganisms in *A. lucorum* treated with medium and low temperatures (T15 and T25) (Figures 1A,B). High temperatures may be beneficial for the symbiosis of more types of microorganisms within the body of *A. lucorum*. According to Simpson’s index, there is not much difference in microorganism species richness at different temperatures (T15T, T25T, and T35T) in no imidacloprid condition (T) (Figure 1C). It is possible that no imidacloprid conditions may have led to greater overall health of *A. lucorum*, resulting in less temperature dependent microbial species diversity. According to Shannon’s index, observe that under high temperature and application of imidacloprid conditions (T35H, T35L), Shannon’s index is higher than that of other groups of samples (Figure 1D). This indicates that with high temperature and imidacloprid, the microbial species diversity in *A. lucorum* is higher. The application of imidacloprid in high-temperature environments led to an increase in the species diversity of microorganisms in the body of *A. lucorum*.

### 3.3 Bacterial community structure is affected by temperature in *A. lucorum* with imidacloprid

**Figure 2:**
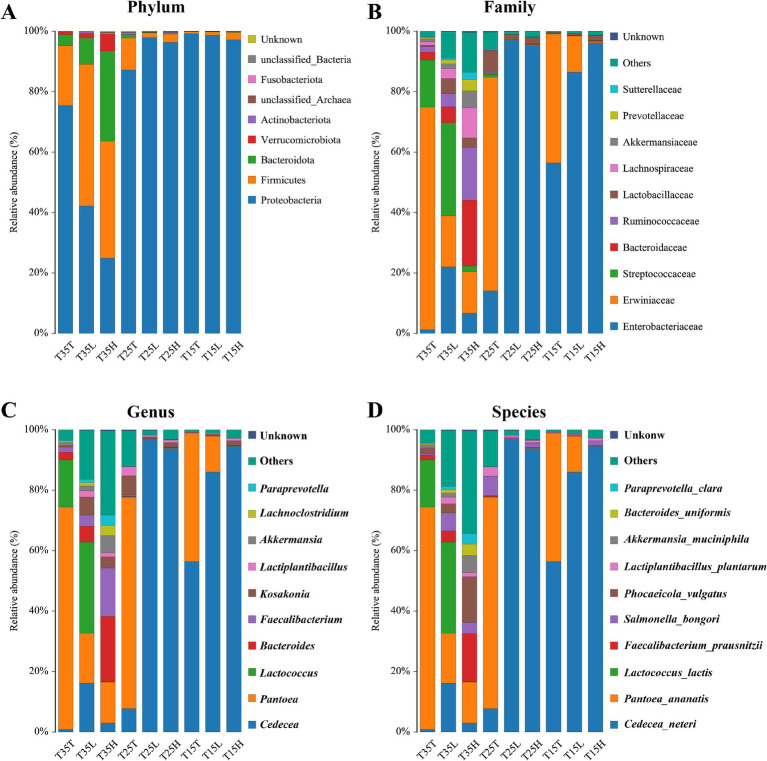
Relative abundance of bacteria communities of *Apolygus lucorum*. **(A)** Relative abundance of bacteria communities at the phylum level in different groups. **(B)** Relative abundance of bacteria communities at the family level in different groups. **(C)** Relative abundance of bacteria communities at the genus level in different groups. **(D)** Relative abundance of bacteria communities at the species level in different groups.

At the phylum classification level, 16S rRNA microbial sequencing identified Proteobacteria, Firmicutes, Bacteroidetes, Verrucota, Actinobacteria, and others (Figure 2A). Except for conditions T35L and T35H, Proteobacteria is the dominant phylum in most samples. But even in these two treatments, Proteus still accounts for a considerable proportion of the total microbial community (T35L = 43.19%, T35H = 24.95%). When treated with the same concentration of imidacloprid, the lower the temperature, the greater the proportion of Proteobacteria (T35 < T25 < T15) (Figure 2A). In addition, at T15 and T35, the higher the concentration of imidacloprid, the smaller the proportion of Proteobacteria (T > L > H) (Figure 2A). The distribution of Firmicutes in temperature and imidacloprid conditions is similar to that of Proteobacteria, indicating that temperature and imidacloprid treatment can affect microbial community composition and abundance at the phylum level (Figure 2A).

Temperature can affect the community composition and abundance of microorganisms at the family level. Increased diversity of bacterial communities due to high temperatures (T35), while at low temperatures (T15), the composition and abundance of microbial communities are more singular (Figure 2B). At low temperature (T15) treatment, Enterobacteriaceae is the dominant family of three samples (T15T, T15L, T15H). And, as the concentration of imidacloprid increases, the proportion of Enterobacteriaceae gradually increases (T15T < T15L < T15H) (Figure 2B). This indicates that at lower temperatures, the concentration of imidacloprid may affect the proportion of Enterobacteriaceae. When the concentration of imidacloprid is the same, the proportion of Enterobacteriaceae in a high temperature condition will be lower than the proportion of Entereobacteriacaea from a low temperature condition (T35T < T15T, T35L < T15L, T35H < T15H) (Figure 2B).

At the level of genus classification, identified genera include *Cedecea*, *Pantoea*, *Lactococcus*, *Bacteroides*, *Faecalibacterium*, *Kosakonia*, *Lactiplantebacillus*, and others (Figure 2C). Temperature can affect the community composition and abundance of microorganisms at the genus level. At high temperatures (T35), the distribution of microorganisms in more genera is more uniform, while at low temperatures (T15), the composition and abundance of microbial communities are more singular (Figure 2C). At low temperature (T15) treatment, *Cedecea* is the dominant genus of three samples (T15T, T15L, T15H) (Figure 2C). As the concentration of imidacloprid increases, the proportion of Cedecea gradually increases (T15T < T15L < T15H) (Figure 2C). This indicates that at lower temperatures, the concentration of imidacloprid may affect the proportion of *Cedecea*, indicating that the symbiotic relationship between *Cedecea* and *A. lucorum* may be strengthened when imidacloprid is applied at low temperatures. For example, when the concentration of imidacloprid is the same, the proportion of *Cedecea* on *A. lucorum* treated at high temperature will be lower than the proportion of *Cedecae* on *A. lucorum* treated at low temperature (T35T < T15T, T35L < T15L, T35H < T15H).

At the species classification level, identified species include *Cedecea neteri*, *Pantoea anatis*, *Lactococcus lactis*, *Faecalibacterium praussnitzii*, *Salmonella bongori*, *Phocaeicola vulgatus*, *Lactobacillus plantarum*, *Akkermansia muciniphila*, *Bacteroides uniformis*, *Paraprevotella Clara*, and others (Figure 2D). Temperature can affect the community composition and abundance of microorganisms at the species level. At high temperatures (T35), the distribution of microorganisms in more species is more uniform, while at low temperatures (T15), the composition and abundance of microbial communities are more singular. At low temperature (T15) treatment, *Cedecea neteri* is the dominant species of three samples (T15T, T15L, and T15H) (Figure 2D). As the concentration of imidacloprid increases, the proportion of *Cedecea neteri* gradually increases (T15T < T15L < T15H). This indicates that at lower temperatures, the concentration of imidacloprid may affect the proportion of *Cedecea neteri*, suggesting that the symbiotic relationship between *Cedecea neteri* and *A. lucorum* may be strengthened when imidacloprid is applied at low temperatures. When the concentration of imidacloprid is the same, the proportion of *Cedecea neteri* treated at high temperature will be lower than the proportion of *Cedecea neteri* when treated at low temperature (T35T < T15T, T35L < T15L, T35H < T15H) (Figure 2D). This indicates that high temperatures may disrupt the symbiotic relationship between *Cedecea neteri* and *A. lucorum*.

### 3.4 RDA/CCA of microbiota at different temperature treatment conditions in *A. lucorum* with imidacloprid

**Figure 3:**
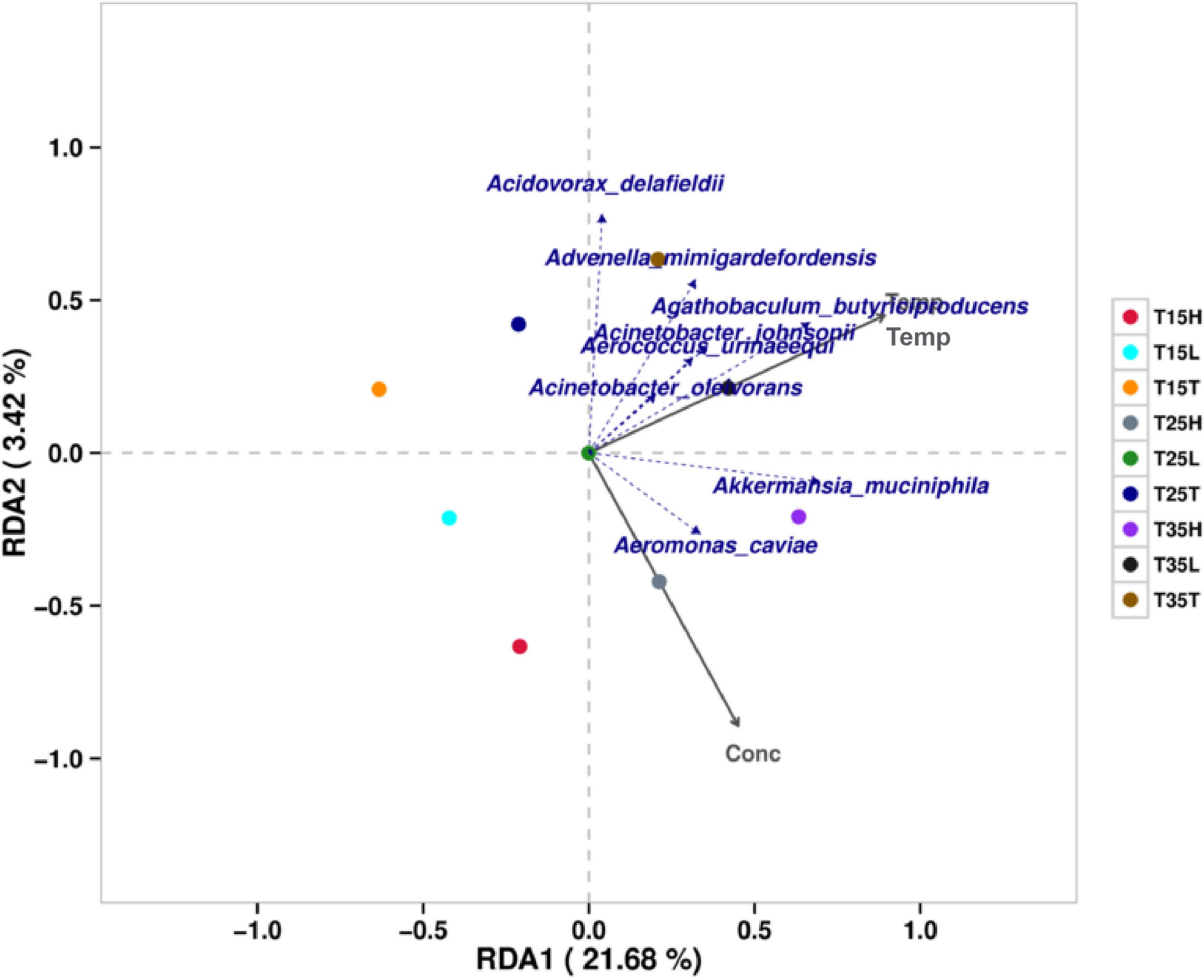
Correlation and association analysis of the bacterial community of *Apolygus lucorum* at the species level under different treatments. (1) The length of the arrow represents the intensity of the impact of the environmental factor on community change. The longer the arrow is, the greater the impact of the environmental factor. (2) The angle between the arrow and the coordinate axis represents the correlation between the environmental factor and the coordinate axis. The smaller the angle, the higher the correlation. (3) The closer the sample point is to the arrow, the stronger the effect of this environmental factor on the sample. (4) If the sample is located in the same direction as the arrow, this indicates a positive correlation between environmental factors and changes in the sample species community. If the sample is located in the opposite direction of the arrow, this indicates a negative correlation between environmental factors and changes in the sample species community.

RDA (redundancy analysis)/CCA (anonical correspondence analysis) analysis was performed for 27 samples using species specific classification. Figure 3 shows the results considering environmental factors temperature and imidacloprid concentration. The results include two grey coordinate axes for temperature (Temp) and imidacloprid concentration (Conc). Microbiological species with highly correlated with temperature are: *Agathobaculum butyriciproducens*, *Acinetobacter johnsonii*, *Aerococcus Ulinaeequi*, *Acinetobacter oleivorans*, *Advenella mimigardefordensis*, and *Akkermansia muciniphila*. The microorganisms that are greatly affected by temperature in the community include *Agathobaculum butyriciproducens*, *Acidovorax delafieldii*, *Advenella migardefenensis*, and *Akkermansia muciniphila*. Microorganisms with a high correlation with imidacloprid concentration include *Aeromonas caviae* and *Akkermansia muciniphila*. The microorganisms that are greatly affected by the concentration of imidacloprid in the community include *Aeromonas caviae* and *Akkermansia muciniphila*.

### 3.5 Function prediction

**Figure 4:**
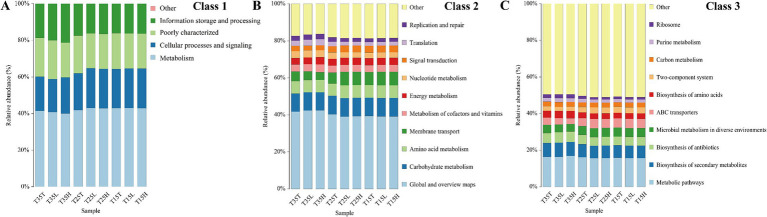
Functional prediction diagram of *Apolygus lucorum* under different treatments. **(A)** Class 1 of the COG annotations. **(B)** Class 2 of the COG annotations. **(C)** Class 3 of the COG annotations.

To understand the differences between these samples, we then performed functional prediction using COG annotation (Figure 4). As shown in the figure, the functions are mainly concentrated in the areas of metabolism, cellular processes and signaling, and information storage and processing. Among them, metabolism accounts for the largest proportion, exceeding 40% in all samples (Figure 4A). Global and overview maps and metabolic pathways were largest in Class 2 and Class 3, respectively (Figures 4B,C). Therefore, bacteria have an important role in *A. lucorum*, and the effects of temperature and imidacloprid treatment on the relative abundance of bacteria should not be ignored.

## 4 Discussion

The 16S rRNA sequencing technique has the potential to enable researchers to build a more accurate and comprehensive understanding of the diversity and richness of microorganisms in the body of *A. lucorum* under different treatments, thereby better utilizing microbial resources for pest control. Our study found that among all treatment groups, Proteobacteria and Firmicutes are dominant phyla, which is similar to the microbial community composition of other insect symbiotic communities (Mason and Raffa, 2014; Chen et al., 2018; Zhao et al., 2019; Gao et al., 2020). The bacterial community structure and diversity associated with the *A. lucorum* varies under different temperature conditions. At high temperatures (T35), the distribution of microorganisms in more species is more uniform, while at low temperatures (T15), the composition and abundance of microbial communities are more singular. This indicates that temperature can affect the community composition and abundance of microorganisms in the body of *A. lucorum* at the species level. This could be due to the complex bidirectional effects between insects and microorganism symbiotes to tolerate and adapt to different thermal environments (Iltis et al., 2022). Interactions between host and microbial symbiotes behavior and physiological activities may be the mechanism of the effects seen in varied temperatures. The thermal environment experienced by microorganisms interacting with the host is influenced by the host’s behavior and physiological activities, such as temperature regulation behavior and microbial environment selection (Arnold et al., 2019; Truitt et al., 2018; Hague et al., 2020).

According to the RDA/CCA analysis, we found that the microbiological species with high temperature correlation are: *Agathobaculum butyriciproducens*, *Acinetobacter johnsonii*, *Aerococcus Ulinaeequi*, *Acinetobacter oleivorans*, *Advenella mimigardefordensis*, *Akkermansia muciniphila*. The microorganisms that are most greatly affected by temperature in the community include *Agathobaculum butyriciproducens*, *Acidovorax delafieldii*, *Advenella migardefenensis*, and *Akkermansia muciniphila*. By analyzing the bacterial community structure at different temperatures in *A. lucorum* with imidacloprid, we found that when the concentration of imidacloprid is the same, the proportion of *Cedecea neteri* from high temperature treatment conditions is lower than that at low temperature treatment conditions (T35T < T15T, T35L < T15L, T35H < T15H). This indicates that high temperatures may disrupt the symbiotic relationship between *Cedecea neteri* and *A. lucorum*. Temperature may affect the symbiosis between microorganisms and insects, thereby affecting the toxicity of pesticides. Studies have shown that elevated temperature can have a negative impact on the symbiosis between *Nilaparvata lugens* and its microbial community leading to increased sensitivity of the host to insecticides such as imidacloprid (Zhang et al., 2021). Studies have shown that temperature does indeed affect the toxicity of imidacloprid. For an insecticide such as imidacloprid, increasing temperatures enhances the acute toxicity of imidacloprid to earthworms *Eisenia fetida* (Velki and Ečimović, 2015). The toxicity of imidacloprid to non-target organisms *E. andrei* and soil flea *Folsomia candidai* increased with increasing temperature in different soils (Bandeira et al., 2020). Using the nymph of *A. lucorum* from Cangzhou, Hebei as the test insect, imidacloprid is a positive temperature effect insecticide for *A. lucorum* (Ma et al., 2012). Different concentrations of imidacloprid were used to treat *A. lucorum*, and their mortality rates were tested. The results showed that the toxicity of imidacloprid to *A. lucorum* increased with increasing temperature (Liu et al., 2016). In the present research we posit that a potential mechanism for this change in toxicity may be due to changes in the amount and composition of the associated microbial community.

According to the RDA/CCA analysis, we found that the microorganisms with a high correlation with imidacloprid concentration include *Aeromonas caviae* and *Akkermansia muciniphila*. The microorganisms that are greatly affected by the concentration of imidacloprid in the community include *Aeromonas caviae* and *Akkermansia muciniphila*. By analyzing the bacterial community structure at different temperatures in *A. lucorum* with imidacloprid, we found that at low temperature (T15) treatment, *Cedecea neteri* is the dominant species for three samples (T15T, T15L, and T15H). And, as the concentration of imidacloprid increases, the proportion of *Cedecea neteri* gradually increases (T15T < T15L < T15H). This indicates that at lower temperatures, the concentration of imidacloprid may affect the proportion of *Cedecea neteri*, suggesting that the symbiotic relationship between *Cedecea neteri* and *A. lucorum* may be strengthened during imidacloprid treatment at low temperatures. These symbiotic bacteria may play an important role in ameliorating insect sensitivity to insecticides. Studies have shown that fungicides may enhance insecticidal capabilities when added to imidacloprid. For example, using specific fungicides as additives to imidacloprid to control *Nilaparvata lugens*, improves insecticide efficacy and may reduce the use of imidacloprid in rice fields (Shentu et al., 2016). Some symbiotic bacteria can also degrade insecticides in the body of pests, such as the detoxification process of organophosphorus insecticide fenitrothion in the body of the cabbage beetle *Riptotus pedestris*. Intestinal symbiotic bacteria degrade fenithrothion into non insecticidal but bactericidal compounds which are then excreted by the host insect. This integrated “host symbiotic detoxification relay” can simultaneously maintain symbiosis and efficient pesticide degradation (Sato et al., 2021). The insect *Riptotus pedestris* contains symbiotic bacteria of the *Burkholderia* genus, which are obtained from soil. The *Burkholderia* strain, which can degrade fenitrothion, has established a specific and beneficial symbiotic relationship with *R. pedestris*, and endows host insects with resistance to fenitrothion (Kikuchi et al., 2012). Aphids infected with *Hamilton* exhibit low sensitivity to low concentrations of insecticides, including imidacloprid (Li et al., 2021). In *B. tabaci*, imidacloprid has a significant impact on strains with dual infections of *Rickettsia arsenopsis* and *Wolbachia arsenopsis*, which also carry a higher number of symbiotic bacteria (Ghanim and Kontsedalov, 2009).

Symbiotic microorganisms can affect the physiology, reproduction, and adaptability of hosts (Werren et al., 2008). At the same time, temperature can affect the host and symbiotes, as well as the interaction between their behavior and physiological activities. The thermal environment experienced by microorganisms interacting with the host is influenced by the host’s behavior and physiological activities, such as temperature regulation behavior and microbial environment selection (Arnold et al., 2019; Truitt et al., 2018; Hague et al., 2020). Therefore, there is a complex bidirectional relationship between insects and symbiotes to tolerate and adapt to different thermal environments (Iltis et al., 2022). Microorganisms typically have high host specificity and persist in the host population (Bennett and Moran, 2015). A growing body of research supports that symbiotic microorganisms contribute to the health and other physiological aspects of their hosts. Symbiotic microorganisms have been demonstrated to assist in immune system regulation, digestion, detoxification, and nitrogen fixation, as well as providing protection against abiotic stress and pathogens (Kaltenpoth et al., 2005; Teixeira et al., 2008; Heyworth and Ferrari, 2015; Abbott et al., 2021).

The aim of this study is to understand the dynamics and diversity of symbiotic bacteria of *A. lucorum* treated with imidacloprid at a range of temperatures. Building a mechanistic understanding of the *A. lucorum* microbiome will support efforts to design important practical applications in the development of microbially based strategies for the management of insect pests (Crotti et al., 2012). Global climate change is an unprecedented challenge we face, with rising temperatures becoming a driving force for biodiversity loss and habitat degradation (McConkey and O’Farrill, 2015). In agriculture, climate change will likely alter many characteristics of pests, which may lead to more severe crop losses. Understanding the response and adaptation of pests to increasing temperatures, as well as identifying and designing new pest management methods, is an important research focus in response to global climate change (Potts et al., 2010; Moran and Alexander, 2014).

Temperature and imidacloprid concentration can affect the microbial community composition and abundance of *A. lucorum*. *Aeromonas caviae* and *Akkermansia muciniphila* have a high correlation with imidacloprid concentration, and the community is greatly affected by imidacloprid concentration. These two species may be key microorganisms that affect the resistance of *A. lucorum* to imidacloprid due to temperature. More research is needed to verify the exact function of these bacteria in these conditions.

## Data Availability

The data presented in the study are deposited in the NCBI repository, accession number PRJNA1185427.

## Footnotes

^1^https://qiime2.org/

^2^https://www.biocloud.net

^3^https://www.ncbi.nlm.nih.gov/COG/
