# Supplementary material for: Symbiotic microbial population composition of Apolygus lucorum under temperature and pesticide pressures
Source: Front Microbiol. 2024 Dec 4;15:1485708. doi: 10.3389/fmicb.2024.1485708 (PMC11656308; doi:10.3389/fmicb.2024.1485708)
Supplement: Supplementary file 1 [file Table_1.docx]

**Table S1** Statistics of sample sequencing data processing results

| Sample ID | Raw CCS | EffectiveCCS | AvgLen(bp) | Effective(%) | |
| --- | --- | --- | --- | --- | --- |
| T15H1 | 6887 | 6871 | 1466 | | 99.77 |
| T15H2 | 5237 | 5223 | 1466 | | 99.73 |
| T15H3 | 6942 | 6922 | 1466 | | 99.71 |
| T15L1 | 8143 | 8098 | 1465 | | 99.45 |
| T15L2 | 4441 | 4421 | 1465 | | 99.55 |
| T15L3 | 6831 | 6802 | 1463 | | 99.58 |
| T15T1 | 7034 | 6988 | 1464 | | 99.35 |
| T15T2 | 7676 | 7623 | 1464 | | 99.31 |
| T15T3 | 7704 | 7634 | 1464 | | 99.09 |
| T25H1 | 8320 | 8286 | 1465 | | 99.59 |
| T25H2 | 5778 | 5751 | 1466 | | 99.53 |
| T25H3 | 6031 | 6008 | 1466 | | 99.62 |
| T25L1 | 7115 | 7095 | 1465 | | 99.72 |
| T25L2 | 8064 | 8056 | 1463 | | 99.90 |
| T25L3 | 7230 | 7221 | 1463 | | 99.88 |
| T25T1 | 5026 | 5002 | 1468 | | 99.52 |
| T25T2 | 4958 | 4934 | 1468 | | 99.52 |
| T25T3 | 5478 | 5429 | 1469 | | 99.11 |
| T35H1 | 3048 | 2836 | 1457 | | 93.04 |
| T35H2 | 4418 | 4131 | 1456 | | 93.50 |
| T35H3 | 5046 | 4635 | 1455 | | 91.85 |
| T35L1 | 6222 | 5942 | 1465 | | 95.50 |
| T35L2 | 5460 | 5360 | 1466 | | 98.17 |
| T35L3 | 5286 | 5229 | 1469 | | 98.92 |
| T35T1 | 7597 | 7534 | 1464 | | 99.17 |
| T35T2 | 7439 | 7374 | 1464 | | 99.13 |
| T35T3 | 8071 | 7990 | 1465 | | 99.00 |

**Table S2** 16s basic information on microbial sequencing of sample

| **Sample** | **Number of valid reads** | **Number of OTUs** | **Number of different taxonomic categories** | | | | | |  |
| --- | --- | --- | --- | --- | --- | --- | --- | --- | --- |
|  |  |  | **Phylum** | **Class** | **Order** | **Family** | **Genus** | **Species** |  |
| T15H1 | 6086 | 55 | 4 | 6 | 17 | 24 | 43 | 53 |  |
| T15H2 | 4660 | 53 | 7 | 9 | 21 | 28 | 43 | 52 |  |
| T15H3 | 6118 | 46 | 5 | 7 | 15 | 21 | 35 | 45 |  |
| T15L1 | 7424 | 38 | 7 | 9 | 18 | 23 | 33 | 36 |  |
| T15L2 | 3269 | 47 | 6 | 8 | 21 | 26 | 41 | 46 |  |
| T15L3 | 6700 | 31 | 4 | 6 | 10 | 15 | 27 | 30 |  |
| T15T1 | 6813 | 37 | 4 | 6 | 17 | 23 | 35 | 36 |  |
| T15T2 | 7386 | 29 | 5 | 6 | 13 | 16 | 26 | 28 |  |
| T15T3 | 7464 | 33 | 4 | 6 | 12 | 18 | 30 | 32 |  |
| T25H1 | 7431 | 58 | 5 | 7 | 19 | 26 | 46 | 56 |  |
| T25H2 | 5015 | 55 | 5 | 7 | 18 | 25 | 44 | 54 |  |
| T25H3 | 5235 | 83 | 6 | 9 | 18 | 33 | 64 | 79 |  |
| T25L1 | 6506 | 48 | 6 | 9 | 20 | 25 | 38 | 46 |  |
| T25L2 | 7917 | 49 | 6 | 8 | 17 | 24 | 40 | 47 |  |
| T25L3 | 6970 | 46 | 6 | 8 | 17 | 22 | 38 | 45 |  |
| T25T1 | 3444 | 76 | 5 | 8 | 22 | 34 | 59 | 74 |  |
| T25T2 | 3613 | 89 | 7 | 10 | 27 | 44 | 70 | 88 |  |
| T25T3 | 3811 | 89 | 7 | 10 | 26 | 39 | 65 | 87 |  |
| T35H1 | 2124 | 130 | 6 | 8 | 24 | 45 | 92 | 120 |  |
| T35H2 | 2555 | 116 | 7 | 9 | 25 | 40 | 79 | 109 |  |
| T35H3 | 3094 | 124 | 6 | 9 | 26 | 41 | 89 | 116 |  |
| T35L1 | 4052 | 154 | 7 | 10 | 30 | 51 | 102 | 144 |  |
| T35L2 | 3916 | 126 | 5 | 8 | 27 | 46 | 92 | 121 |  |
| T35L3 | 3883 | 103 | 6 | 9 | 26 | 39 | 74 | 99 |  |
| T35T1 | 6975 | 96 | 5 | 8 | 21 | 32 | 67 | 91 |  |
| T35T2 | | 6997 | 87 | 5 | 7 | 20 | 33 | 63 | 82 |
| T35T3 | | 6957 | 106 | 5 | 7 | 21 | 36 | 75 | 99 |
| Total | | 146415 | 180 | 8 | 11 | 33 | 60 | 117 | 168 |

**Table S3** Alpha diversity index statistics

| **Sample ID** | | **Feature** | | **ACE** | | **Chao1** | | **Simpson** | | **Shannon** | | **Coverage** | |
| --- | --- | --- | --- | --- | --- | --- | --- | --- | --- | --- | --- | --- | --- |
| T15H1 | 55 | | 80.1846 | | 72.5 | | 0.0984 | | 0.5089 | | 0.9965 | |  |
| T15H2 | 53 | | 98.9736 | | 72.25 | | 0.1249 | | 0.6138 | | 0.9953 | |  |
| T15H3 | 46 | | 63.6218 | | 63 | | 0.099 | | 0.4928 | | 0.9972 | |  |
| T15L1 | 38 | | 102.0353 | | 80 | | 0.24 | | 0.7267 | | 0.9972 | |  |
| T15L2 | 47 | | 70.3333 | | 64.5 | | 0.2846 | | 0.9772 | | 0.9936 | |  |
| T15L3 | 31 | | 57.4393 | | 52.8571 | | 0.233 | | 0.6635 | | 0.9973 | |  |
| T15T1 | 37 | | 49.5203 | | 46.5455 | | 0.5048 | | 1.1397 | | 0.9978 | |  |
| T15T2 | 29 | | 113.2111 | | 42.3333 | | 0.4996 | | 1.0738 | | 0.9978 | |  |
| T15T3 | 33 | | 50.9486 | | 48 | | 0.4987 | | 1.1051 | | 0.9979 | |  |
| T25H1 | 58 | | 77.2192 | | 77 | | 0.0902 | | 0.4763 | | 0.9973 | |  |
| T25H2 | 55 | | 70.6048 | | 63 | | 0.1219 | | 0.612 | | 0.9968 | |  |
| T25H3 | 83 | | 144.6116 | | 120.7143 | | 0.1543 | | 0.8172 | | 0.9937 | |  |
| T25L1 | 48 | | 60.6364 | | 55.5556 | | 0.061 | | 0.3355 | | 0.9974 | |  |
| T25L2 | 49 | | 66.7562 | | 79.6 | | 0.064 | | 0.3572 | | 0.9977 | |  |
| T25L3 | 46 | | 62.1668 | | 58.3636 | | 0.0648 | | 0.3445 | | 0.9976 | |  |
| T25T1 | 76 | | 104.3406 | | 101 | | 0.4695 | | 1.9805 | | 0.9925 | |  |
| T25T2 | 89 | | 108.6069 | | 108.1176 | | 0.4682 | | 2.014 | | 0.9928 | |  |
| T25T3 | 89 | | 109.6528 | | 104.8125 | | 0.5542 | | 2.3998 | | 0.994 | |  |
| T35H1 | 130 | | 159.3633 | | 167.7143 | | 0.9469 | | 5.3047 | | 0.9845 | |  |
| T35H2 | 116 | | 134.5377 | | 132.6667 | | 0.9174 | | 4.6651 | | 0.9902 | |  |
| T35H3 | 124 | | 135.0527 | | 133.5 | | 0.9204 | | 4.7721 | | 0.9939 | |  |
| T35L1 | 154 | | 170.8814 | | 175 | | 0.9296 | | 4.9516 | | 0.9931 | |  |
| T35L2 | 126 | | 147.8991 | | 143.6522 | | 0.8224 | | 3.6391 | | 0.9926 | |  |
| T35L3 | 103 | | 132.5846 | | 154 | | 0.7039 | | 2.7281 | | 0.9912 | |  |
| T35T1 | 96 | | 122.9213 | | 112.9167 | | 0.4255 | | 1.6173 | | 0.9958 | |  |
| T35T2 | 87 | | 101.2278 | | 101 | | 0.4371 | | 1.5921 | | 0.997 | |  |
| T35T3 | 106 | | 155.5256 | | 147.0526 | | 0.4364 | | 1.6718 | | 0.9943 | |  |


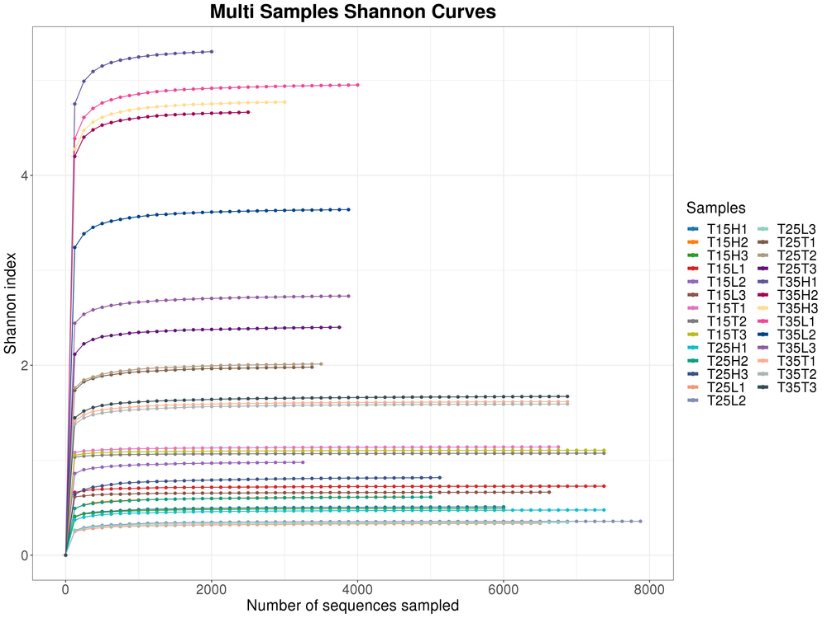


**Fig. S1** Dilution curve of shannon diversity index of *Apolygus lucorum* under different treatments.
